# Chemical Profiling and Authentication of *Boswellia* Species and Frankincense Products: Advances in Metabolomics and Quality Markers

**DOI:** 10.3390/plants15192916

**Published:** 2026-09-23

**Authors:** Nataša Nastić, Kristian Pastor

**Affiliations:** 1 Department of Chemical and Petroleum Engineering, Khalifa University of Science and Technology, Abu Dhabi P.O. Box 127788, United Arab Emirates; 2 Faculty of Technology Novi Sad, University of Novi Sad, 21000 Novi Sad, Serbia

**Keywords:** *Boswellia*, frankincense, olibanum, chemical profiling, authentication, metabolomics, chemometrics, quality markers, boswellic acids, essential oils, adulteration detection

## Abstract

Frankincense is an aromatic oleo-gum resin obtained from *Boswellia* species and is widely used in traditional medicine, pharmaceuticals, nutraceuticals, cosmetics, perfumery, and incense products. Its expanding trade has intensified concerns over species substitution, geographical mislabeling, adulteration, compositional variability, and inconsistent product quality. Authentication is complicated by an extensive overlap in volatile and non-volatile metabolites among commercially important species and by variation arising from provenance, environment, harvesting, storage, processing, and extraction. This review examines chemical profiling and authentication strategies for *Boswellia* resins and frankincense-derived products, focusing on volatile terpenes, diterpenoids, boswellic acids, methoxyalkanes, stereochemical signatures, and multivariate quality markers. The available evidence shows that single markers and nonspecific total-acid assays are insufficient for robust authentication, whereas orthogonal workflows integrating targeted quantification, chemical fingerprints, stereochemical information, and validated multivariate models provide substantially greater confidence. Future progress depends on authenticated reference collections, standardized sampling and analytical protocols, certified reference materials, expanded spectral libraries, interlaboratory validation, explainable machine learning, and traceable supply-chain metadata.

## 1. Introduction

Frankincense, also known as olibanum, is an aromatic oleo-gum resin obtained from trees of the genus *Boswellia* (family Burseraceae). For more than 5000 years, frankincense has been traded as one of the world’s most valuable natural products and has played a central role in religious ceremonies, traditional medicine, perfumery, cosmetics, and, more recently, in the pharmaceutical and nutraceutical industries [1,2,3]. Its historical importance is reflected in the designation of Oman’s Land of Frankincense as a UNESCO World Heritage property in 2000. UNESCO describes frankincense production and distribution as one of the most important luxury trades of the ancient world and recognizes the archeological sites and *Boswellia sacra* stand of Dhofar as outstanding evidence of this long-standing trade [4]. Today, frankincense-derived products including raw resin, essential oils, solvent extracts, dietary supplements, and cosmetic formulations, represent an expanding global market driven by increasing consumer demand for natural health products and plant-based therapeutics [2,5].

The genus *Boswellia* comprises approximately 25 accepted species distributed mainly across the Arabian Peninsula, tropical Africa, and the Indian subcontinent [6]. Among these, *B. sacra* Flück., *B. carteri* Birdw., *B. serrata* Roxb., *B. papyrifera* (Delile) Hochst., and *B. frereana* Birdw. are the principal commercial sources of frankincense [7]. These species differ considerably in their geographical distribution, resin characteristics, chemical composition, biological activities, and commercial value, making accurate botanical identification essential for ensuring product quality, efficacy, and authenticity [8]. Commercially available frankincense is typically graded according to quality in traditional markets, with *Najdi*, *Sharzi*, and *Shaabi* representing high-, medium-, and low-quality grades, respectively. The biological and commercial significance of frankincense is closely associated with its chemical diversity. Frankincense resin contains hundreds of metabolites belonging primarily to mono-, sesqui-, di-, and triterpenoids together with polyphenols, essential oils, tannins, alkaloids, saponins and other compounds [8,9]. The volatile fraction is dominated by monoterpenes such as α-pinene, β-pinene, limonene, myrcene, sabinene, and terpinen-4-ol, while the non-volatile fraction is characterized by pentacyclic triterpenoids, particularly α- and β-boswellic acids, 11-keto-β-boswellic acid (KBA), and 3-O-acetyl-11-keto-β-boswellic acid (AKBA), which are widely regarded as the principal bioactive constituents responsible for many of the reported anti-inflammatory, anticancer, antioxidant, antimicrobial, and neuroprotective properties of frankincense [10,11].

Despite its long history of use, the authentication of frankincense has become an important issue due to the rapid expansion of international trade, increasing market value, and growing demand for botanical supplements. Recent analyses indicate that the global trade in frankincense resin exceeds 15,000 tons annually, with *B. papyrifera* representing the largest traded species (approximately 3500–7900 tons year^−1^), followed by *B. sacra*, *B. serrata*, and several Somali species including *B. carteri*, *B. frereana*, *B. rivae*, and *B. neglecta* [12]. Commercial frankincense products are frequently affected by species substitution, geographical mislabeling, variable harvesting practices, inconsistent processing, and deliberate adulteration with cheaper *Boswellia* species, other plant resins, vegetable oils, synthetic fragrance compounds, or isolated terpenes [13]. Such practices not only affect product quality and therapeutic efficacy, but also present challenges for regulatory authorities, manufacturers, researchers, and consumers. The complexity of frankincense chemistry, together with the overlap in metabolite composition among *Boswellia* species, has led to the development of sophisticated analytical approaches for authentication and quality assessment. Among the analytical advances, chemical fingerprinting has emerged as one of the most powerful strategies for the authentication of frankincense. Rather than relying on individual marker compounds, fingerprinting approaches integrate the entire metabolite profile to distinguish species, detect adulteration, identify geographical origin, evaluate processing methods, and assess commercial quality [14]. Recent studies have demonstrated that enantiomeric distributions of monoterpenes, characteristic diterpenes, species-specific methoxyalkanes, and quantitative profiles of boswellic acids can serve as robust markers for differentiating closely related *Boswellia* species and identifying adulterated commercial products [13,15,16]. Notably, comparative analyses of commercial and authenticated *B. carteri* essential oils have revealed adulteration involving synthetic limonene, castor oil, copaiba oleoresin, phthalates, and substitution with other *Boswellia* species, highlighting the need for rigorous authentication protocols throughout the supply chain [13].

Numerous studies have investigated the phytochemistry, pharmacology, essential oil composition, and therapeutic applications of individual *Boswellia* species, while comprehensive evaluations of chemical profiling methodologies, authentication strategies, marker compounds, chemometric approaches, and analytical workflows across different frankincense products are still lacking. Therefore, the objective of this review is to provide a comprehensive overview of the current state of chemical profiling and the authentication of *Boswellia* species and frankincense-derived products. This review discusses the phytochemical diversity of commercially important *Boswellia* species, evaluates the analytical platforms used for chemical characterization, summarizes chemical markers for species and authentication, examines current approaches for adulteration detection and quality control, and highlights emerging developments in metabolomics, chemometrics, and artificial intelligence that are expected to shape the next generation of botanical authentication strategies.

### Literature Search and Review Methodology

A literature search was conducted in Scopus, PubMed, and Google Scholar from database inception to 28 July 2026, with no lower publication-date limit. Search strings combined “*Boswellia*” OR “frankincense” OR “olibanum” with “chemical profiling”, “metabolomics”, “chemotaxonomy”, “authentication”, “adulteration”, “quality marker”, “GC-MS”, “LC-MS”, “HPLC”, “NMR”, “NIR”, “DART-MS”, “MALDI-TOF”, or “chemometrics”. The syntax and search fields were adapted to the requirements of each database. Only publications written in English were considered. Peer-reviewed original studies and reviews addressing the taxonomy, chemical composition, analytical characterization, authentication, adulteration, or quality control of *Boswellia* resins and derived products were considered. Authoritative taxonomic databases, pharmacopeial monographs, and reports from recognized international organizations were included where relevant. Studies were excluded when botanical identity or analytical methodology was insufficiently described, the material was unrelated to *Boswellia* or frankincense, or the publication did not contribute evidence relevant to chemical profiling or authentication. Because the purpose was to provide a broad critical synthesis rather than a formal systematic review or meta-analysis, no quantitative evidence synthesis was undertaken.

## 2. Botanical Origin, Taxonomy and Commercial Importance

The genus *Boswellia* Roxb. ex Colebr. belongs to the family Burseraceae, a pantropical family comprising approximately 19 genera and more than 700 species of resin-producing trees and shrubs, including the economically important genera *Boswellia*, *Commiphora*, *Canarium*, and *Bursera* [17]. Members of the family are characterized by the presence of resin canals that produce aromatic oleo-gum resins in response to mechanical injury. Resin secretion is induced when the bark of *Boswellia* species is damaged by herbivores or insects or intentionally cut prior to harvesting. These oleo-gum resins function as an effective defense mechanism by deterring herbivores, inhibiting insect infestation and microbial infection, and minimizing water loss under arid environmental conditions [18].

Currently, approximately 25 *Boswellia* species are recognized, although the taxonomy of the genus has long been challenging because of high morphological variability, overlapping diagnostic characters among species, historical misidentifications, and inconsistent nomenclature [19]. Most species are endemic to the arid and semi-arid regions of northeastern Africa, southern Arabia, and the Indian subcontinent, where they occur in fragmented populations on rocky limestone escarpments, dry woodlands, and mountainous landscapes (Figure 1) [19].

Only a limited number of species contribute substantially to international frankincense production. These include *B. sacra*, *B. carteri*, *B. frereana*, *B. papyrifera*, and *B. serrata*, while *B. neglecta*, *B. rivae*, and *B. occulta* enter local or niche markets but are not major contributors to international frankincense production [7]. The taxonomic relationship between *B. sacra* and *B. carteri* has long been debated. Traditionally, the two taxa have often been treated as conspecific because of their morphological overlap and similar phytochemical profiles, leading many authors to regard *B. carteri* as a synonym or regional variant of *B. sacra* [20]. Under this interpretation, populations from southern Arabia were assigned to *B. sacra*, whereas morphologically similar populations from Somalia were referred to as *B. carteri*. Current taxonomic authorities, however, treat *B. carteri* as a synonym of the accepted name *B. sacra*, whose native distribution includes Somalia, Oman, and Yemen [6,19,20,21,22]. Recent integrative taxonomic evidence combining morphological, molecular, and chemical data supports the recognition of *B. carteri* and *B. sacra* as distinct species [23,24]. However, in this review when discussing individual studies or commercial products, the name reported in the original source is retained because the identity of those materials cannot be retrospectively reassigned. Such usage should not be interpreted as recognition of *B. carteri* and *B. sacra* as separate accepted species. For the same reason, results reported under the two names are presented separately in some sections and tables according to their stated geographical origins and source publications. The major commercial species, their geographical distribution, principal applications, and conservation status are summarized in Table 1.

Frankincense obtained from different *Boswellia* species differs considerably in physical appearance, resin yield, essential oil composition, triterpenoid profile, and commercial value. *B. sacra* resin from Oman is widely regarded as one of the highest-quality frankincense products because of its monoterpene-rich essential oil and characteristic aroma [30]. In contrast, *B. frereana* (“Maydi”) is particularly valued as a chewing resin and in perfumery owing to its pleasant flavor and aroma, and because its resin contains negligible amounts of boswellic acids compared with other commercial *Boswellia* species [31]. *B. serrata* is the principal source of pharmaceutical frankincense extracts because of its high concentration of bioactive pentacyclic triterpenoids, particularly AKBA [32]. In contrast, *B. papyrifera* is the principal source of African frankincense in international trade owing to its extensive natural distribution across Ethiopia, Eritrea, and Sudan as well as its large-scale commercial resin production [33].

Recent taxonomic studies have further expanded the recognized diversity of commercial frankincense. The description of *B. occulta* in 2019 demonstrated that significant quantities of resin entering international markets had previously been incorrectly identified as *B. carteri* [16]. Unlike other commercial species, *B. occulta* produces characteristic methoxyalkanes that provide highly distinctive chemical markers, illustrating how phytochemical profiling can support taxonomic revision and improve species authentication [16,34].

The economic importance of *Boswellia* extends well beyond resin production. Frankincense harvesting represents a major source of income for rural communities throughout the Horn of Africa and southern Arabia, where thousands of households depend on seasonal resin collection [35]. Nevertheless, most commercial resin continues to be harvested from wild populations rather than cultivated plantations, increasing vulnerability to overexploitation and supply-chain variability. Consequently, sustainable resource management and reliable species authentication have become closely interconnected issues. From an authentication perspective, the taxonomic complexity of *Boswellia* presents a great analytical challenge. Resin morphology alone is frequently insufficient for reliable species identification because color, transparency, and tear size are influenced by environmental conditions, harvesting practices, and storage. Likewise, many volatile terpenes are shared among several species, requiring comprehensive chromatographic fingerprints, multivariate chemometric analyses, or complementary molecular approaches to achieve reliable discrimination [36]. These challenges have increased the use of integrated authentication methods combining botanical taxonomy, metabolomics, high-resolution mass spectrometry, spectroscopy, and machine learning.

## 3. Phytochemical Diversity

The remarkable commercial, medicinal, and cultural value of frankincense is largely attributable to its highly diverse phytochemical composition. Frankincense resin is a complex oleo-gum resin consisting of three major fractions: a volatile essential oil fraction (approximately 5–7%), an alcohol-soluble resin fraction (60–80%), and a water-soluble gum fraction (15–20%), although the relative proportions vary considerably among *Boswellia* species, geographical origin, harvesting season, environmental conditions, and post-harvest processing (Figure 2) [37].

Recent phytochemical studies have shown that *Boswellia* species produce hundreds of structurally diverse secondary metabolites, including more than 300 volatile constituents and over 100 diterpenes and triterpenoids, in addition to polysaccharides and minor phenolic compounds [38]. Major chemical constituents in *Boswellia* species are summarized in Table 2.

### 3.1. Volatile Constituents

The volatile fraction of frankincense is responsible for its characteristic aroma and is composed predominantly of diterpenes, monoterpenes (in hydrocarbons, alcohols, and ketonic forms), and sesquiterpenes. Gas chromatography–mass spectrometry (GC-MS) studies have identified more than 300 volatile constituents across the genus, with α-pinene generally representing the dominant compound in most commercially important species [59].

The chemical composition of essential oils differs markedly among *Boswellia* species. The essential oils of *B. sacra* are generally monoterpene-rich and commonly contain α-pinene, α-thujene, limonene, sabinene, β-pinene, myrcene, p-cymene and (E)-β-ocimene [2,30]. Although α-pinene concentrations of approximately 30–70% have been reported in several oils, authenticated samples demonstrate substantial variation [59]. For example, an authenticated Omani *B. sacra* oil was dominated by (E)-β-ocimene and limonene rather than α-pinene [30]. More recent comparisons of cultivated, wild-harvested and commercial Omani samples have also identified additional compositional patterns, including δ-3-carene/α-pinene-rich oils, further indicating that *B. sacra* cannot be represented by a single fixed volatile profile [40]. A broadly similar monoterpene-rich profile is observed in *B. carteri*, in which α-pinene is commonly the principal constituent, accompanied by α-thujene, limonene, δ-3-carene, sabinene, β-pinene, myrcene, and p-cymene [13]. Interpretation of these data requires taxonomic caution because *B. carteri* is frequently treated as synonymous with, or commercially substituted for, *B. sacra* [31]. Analysis of authenticated and commercial *B. carteri* oils has reported α-pinene concentrations of approximately 24–46%, although substantial compositional variation and adulteration have been observed in commercial products [13]. In contrast, *B. serrata* oils are frequently distinguished by high concentrations of α-thujene and other monoterpene hydrocarbons. Commercial Indian resin samples have been reported to contain α-thujene at approximately 61–70%, whereas oils obtained from wild populations contained greater proportions of oxygenated monoterpenes and sesquiterpenes, including α-terpineol, terpinyl esters and eudesmol-type compounds [60]. Other studies have reported α-pinene-dominant *B. serrata* oils, indicating the occurrence of geographical and processing-related chemotypic variation [56]. A recent comparison also found that a commercial oil was dominated by α-thujene, whereas an oil hydrodistilled from commercially obtained resin was dominated by octyl acetate, further illustrating variation among products and sample types [61]. In contrast to the α-pinene- or α-thujene-rich profiles of several other species, *B. papyrifera* is distinguished by high concentrations of the aliphatic ester octyl acetate and its corresponding alcohol, 1-octanol [59]. Authenticated single-tree analyses have confirmed the predominance of these compounds, together with variable quantities of limonene, (E)-β-ocimene and diterpenoids such as cembrene A, incensole derivatives and verticilla-4(20),7,11-triene [53]. The oils of the East African species *B. neglecta* and *B. rivae* are also dominated by monoterpenes, but exhibit distinct multicomponent profiles. *B. neglecta* commonly contains α-pinene, α-thujene, terpinen-4-ol, p-cymene, β-pinene and limonene [52]. Comparative work by Bekana et al. [52] described α-pinene-rich fractions in *B. rivae*. However, a more recent multi-sample investigation of *B. rivae* resin demonstrated a broader compositional range, including variable quantities of α-pinene, α-thujene, β-pinene, δ-3-carene, p-cymene, limonene, β-phellandrene, trans-pinocarveol and trans-verbenol [28]. *B. frereana* essential oils are characterized by moderate-to-high concentrations of α-thujene and highly variable concentrations of α-pinene, accompanied by sabinene, p-cymene, β-pinene and, in some samples, α-phellandrene and α-phellandrene-derived dimers [51]. The recently described *B. occulta* exhibits a distinctive volatile profile dominated by long-chain methoxyalkanes, particularly 1-methoxydecane and 1-methoxyoctane, together with 1-methoxynonane, 1-methoxyundecane, serratol and 4,10-di-epi-guaiol. These compounds have not been reported from other commercially important *Boswellia* species and therefore provide highly specific chemotaxonomic markers [16,34].

### 3.2. Non-Volatile Constituents

The resin fraction contains a wide range of triterpenoids, diterpenoids, and other non-volatile metabolites that largely determine the biological activity of frankincense. Pentacyclic triterpenoids, known as boswellic acids, represent the most investigated constituents owing to their anti-inflammatory, anticancer, antimicrobial, immunomodulatory, and neuroprotective properties [62].

More than twenty naturally occurring boswellic acid derivatives have been identified, with α-boswellic acid (α-BA), β-boswellic acid (β-BA), acetyl-α-boswellic acid (AαBA), acetyl-β-boswellic acid (AβBA), KBA, and AKBA regarded as the principal bioactive molecules [3,62].

The qualitative and quantitative composition of non-volatile constituents varies considerably among *Boswellia* species. *B. serrata* generally contains the highest concentrations of pentacyclic triterpenoids, particularly KBA and AKBA, and is therefore the preferred raw material for standardized medicinal extracts [59]. *B. sacra* and *B. carteri* exhibit broadly similar triterpenoid profiles dominated by α-BA, β-BA, AαBA, AβBA, KBA, and AKBA, although authenticated comparative studies have demonstrated quantitative differences among populations and commercial samples, reflecting geographical origin and harvesting conditions [3,44,63]. In contrast, *B. papyrifera* contains comparatively low concentrations of boswellic acids, but is enriched in diterpenoids, including incensole, incensole acetate, cembrenes, and verticillane-type diterpenes [15]. Similarly, *B. frereana* contains only trace amounts of boswellic acids and is characterized by a predominance of diterpenoid constituents, accounting for its distinct phytochemical profile and comparatively limited use in standardized medicinal preparations [51].

Information on the non-volatile composition of *B. neglecta* and *B. rivae* remains comparatively limited. Available phytochemical studies have identified α-boswellic acid together with triterpenoids such as α-amyrin, β-amyrin, α-amyrenone, and β-amyrenone in both species [52]. Quantitative analyses further indicate that *B. neglecta* and *B. rivae* contain substantially lower concentrations of keto- and acetylketo-boswellic acids than *B. sacra* and *B. serrata*.

### 3.3. Chemotaxonomic Marker Compounds

The identification of robust chemotaxonomic markers is fundamental for the authentication, quality control and traceability of frankincense and frankincense-derived products. Marker compounds should exhibit high discriminatory power, analytical reproducibility and relative stability during harvesting, storage and processing, while showing limited intraspecific variation. However, because the chemical composition of *Boswellia* resins is influenced by genetic, environmental and technological factors, no single metabolite can discriminate all species across their geographical ranges. Consequently, authentication strategies rely on combinations of marker compounds together with multivariate chemical fingerprints rather than individual constituents [36,59].

Among the *Boswellia* fractions, the resin contains the highest concentration of taxonomically informative terpenoids, whereas the essential oil contributes additional volatile markers that are particularly useful for differentiating frankincense species. Pentacyclic triterpenoids, mainly AKBA, KBA, α-BA, and β-BA, represent the principal chemotaxonomic markers of *Boswellia*. Among them, AKBA and KBA are considered the most characteristic and biologically significant constituents because of their restricted occurrence within *Boswellia* and their consistent use as analytical markers in pharmacopeial quality control [15,44]. *B. serrata*, a pharmacopeial Indian frankincense, typically contains the highest concentrations of pentacyclic boswellic acids, particularly AKBA, KBA, β-BA, and acetylated derivatives, which serve as its defining chemical signature. Quantitative determination of AKBA has become a standard criteria for evaluating *B. serrata* extracts used in pharmaceutical preparations [15,62].

In contrast, Arabian and East African species such as *B. sacra* and *B. carteri* exhibit comparatively lower levels of boswellic acids, but are enriched in diterpenoid cembranoids, including incensole, incensole acetate, incensole oxide, and related oxygenated derivatives. These diterpenes are regarded as characteristic chemotaxonomic markers for the “true frankincense” species and distinguish them from *B. serrata* [62]. Both species generally exhibit α-pinene-rich essential oils. *B. papyrifera* is consistently distinguished by high proportions of octyl acetate and 1-octanol, which remain among the most reliable volatile markers for this species [52].

Recent chemotaxonomic studies have expanded to include East African species. Authenticated *B. neglecta* is characterized by α-pinene-rich essential oils containing relatively high levels of terpinen-4-ol and verbenone, whereas *B. rivae* exhibits an α-pinene-dominated profile enriched in limonene, p-cymene, trans-verbenol, and α-campholene aldehyde, together with comparatively low concentrations of boswellic acids [28,52]. Likewise, *B. frereana* exhibits a characteristic volatile profile dominated by α-thujene and α-pinene with substantial inter-tree variation and only trace amounts or complete absence of the major boswellic acids [52]. The discovery of methoxyalkanes as unique constituents of *B. occulta* represents one of the most significant advances in *Boswellia* chemotaxonomy during the past decade, providing highly reliable markers for distinguishing this species from morphologically similar *B. carteri* [16,29].

## 4. Factors Influencing Chemical Composition

The chemical composition of frankincense is influenced by a complexity of genetic, environmental, physiological, and technological factors. Consequently, the chemical profile of a resin sample reflects not only its botanical origin, but also geographical location, ecological conditions, harvesting practices, resin age, storage conditions, and extraction methodology. Understanding these sources of variation is essential because they may significantly influence chemical fingerprints and, if not properly considered, lead to erroneous conclusions regarding species identity or product authenticity. Therefore, authentication protocols should distinguish between natural chemical variability and changes resulting from adulteration or species substitution.

### 4.1. Species and Genetic Background

Botanical species represent the primary determinant of frankincense chemistry. Although commercially important *Boswellia* species share numerous volatile and non-volatile constituents, each species possesses characteristic qualitative and quantitative metabolite profiles resulting from differences in terpene and triterpenoid biosynthesis.

Genetic variation among individual trees represents an additional source of heterogeneity. DeCarlo et al. [53] analyzed resin collected separately from 11 authenticated *B. papyrifera* trees growing within the same region of Sudan and reported octyl acetate concentrations ranging from 49.5% to 81.0%, while total diterpenoids varied from 6.6% to 32.7%. Because the trees were sampled from a common geographical location under broadly similar environmental conditions, these differences were attributed primarily to intrinsic genetic variability among individual trees. Comparable findings have been reported for *B. frereana*. Johnson et al. [51] examined resin from twelve authenticated individual trees and observed variation in volatile composition, with α-thujene ranging from 14.5% to 43.9% and α-pinene from 3.0% to 63.0%. In contrast, the triterpenoid fraction exhibited lower variability and was consistently dominated by lupeol, α-amyrin, β-amyrin, and 3-epi-lupeol. These findings demonstrate that individual-tree variation can remain substantial even when species, collection area and general environmental conditions are controlled.

### 4.2. Geographical Origin and Environmental Conditions

Geographical origin significantly influences the chemical composition of *Boswellia* resin through the combined effects of climate, soil characteristics, altitude, rainfall, solar radiation, and water availability [33,46]. Environmental factors affect secondary metabolism by regulating the expression of genes involved in terpene biosynthesis and by modifying carbon allocation toward defensive metabolites [60].

Regional differences in resin chemistry have been documented in several *Boswellia* species. Studies have demonstrated significant geographic variation in the essential oil composition of *B. sacra* resin within the Dhofar region of Oman. The relative abundances of major constituents, including α-pinene, limonene, myrcene, and sabinene, differ markedly among collection sites [64]. Analysis of *B. sacra* resin from coastal and dry mountainous populations in Oman revealed differences in boswellic acid concentrations, with trees growing in dry mountain habitats accumulating higher levels of total boswellic acids than coastal populations [46]. DeCarlo et al. [40] compared cultivated trees, wild individual trees, and commercial wild-harvested materials from southern Oman. The cultivated and wild-derived oils showed comparable overall phytochemical complexity, but a previously unreported δ-3-carene/α-pinene chemotype was detected among individual wild trees. These findings indicate that even within a relatively restricted geographical region, local populations may contain substantial and previously unrecognized phytochemical diversity. Ethiopian *B. papyrifera* populations have been reported to produce essential oils dominated by octyl acetate and n-octanol [52], while Sudanese populations also exhibit octyl acetate-rich profiles but show considerable inter-tree variation in the relative abundance of major and minor volatile constituents [53]. This overlap in the dominant constituents suggests that resin chemistry is influenced more strongly by local environmental conditions than by geographic boundaries.

Climate, particularly rainfall and temperature, strongly influences resin production. Resin yield depends on the tree’s physiological condition established during the preceding rainy season, as adequate rainfall supports growth, photosynthesis, carbohydrate accumulation, and wound healing, whereas prolonged drought reduces carbon assimilation, growth, and the capacity for sustained resin production [33,65]. In addition to regulating resin productivity, climatic conditions can affect resin chemistry. Precipitation and temperature were identified as significant environmental drivers of the volatile organic compound composition of *Boswellia* resin, affecting the relative abundance of monoterpenes and sesquiterpenes across different habitats and seasons [66].

Site-specific environmental factors, including altitude, topography, and soil characteristics, play an important role in the chemical composition of *Boswellia* species. These trees typically inhabit rocky slopes, limestone escarpments, and shallow, nutrient-poor soils where water availability is limited, and drought stress is frequent. However, the edaphic conditions vary considerably among species. For example, *B. sacra* predominantly grow on calcareous limestone substrates in Oman, whereas *B. serrata* is associated with granitic and sandstone-derived soils in India, and *B. papyrifera* is found mainly on volcanic soils in Ethiopia and Eritrea. Such differences in soil mineral composition, water-holding capacity, and nutrient availability are known to influence plant nutritional status and secondary metabolism and may contribute to the interspecific and intraspecific variation observed in terpene and boswellic acid profiles among *Boswellia* species [67].

### 4.3. Harvesting Practices and Storage

Frankincense resin is produced as a defense response following deliberate wounding of the bark. Direct molecular evidence for this wound response has been obtained in *B. serrata*. Transcriptomic and metabolomic analyses showed that mechanical wounding activated terpene-synthase genes and increased the production of volatile resin [68]. Consequently, the frequency, depth and timing of tapping can influence both resin yield and chemical composition by affecting the metabolic allocation of terpenoids and resin acids [69].

Unsustainable harvesting practices involving excessive tapping or insufficient recovery periods not only reduce tree vitality, but they also affect resin production and physiology. In *B. sacra*, resin production increased during the first several tapping rounds before declining with continued tapping [70]. In *B. papyrifera*, resin yield reached its maximum level after several tapping rounds, whereas continuously tapped trees produced lower-quality resin than trees allowed to recover between harvesting seasons [25]. Physiological stress associated with repeated wounding may affect the concentrations of several volatile terpenes and triterpenoids, potentially affecting analytical fingerprints. The harvest season also influences frankincense resin composition. Fresh resin collected during the early tapping rounds often differs chemically from resin obtained during later harvests because resin maturation, oxidation, and the gradual loss of volatile terpenes continue throughout the production season [71].

Following collection, frankincense resin undergoes continuous chemical changes during storage and transportation, primarily due to oxidation and the loss of volatile terpenes through evaporation, resulting in modification of its aroma and overall chemical composition [1]. Essential oil constituents such as α-pinene, limonene and myrcene are particularly sensitive to oxidative degradation, whereas prolonged exposure to heat, oxygen and ultraviolet radiation accelerates the formation of oxygenated terpenoids and resin oxidation products [1,72]. Mathe et al. [73] compared archeological and modern frankincense samples by GC-MS. Ancient samples had lost most monoterpenes through long-term volatilization and oxidation, whereas non-volatile diterpenes and triterpenoid markers remained detectable.

### 4.4. Extraction Method

Grinding, milling and extraction procedures further influence chemical composition by increasing surface area and promoting volatilization of low-molecular-weight compounds. Hydrodistillation, steam distillation, solvent extraction, Soxhlet extraction, ultrasound-assisted extraction, microwave-assisted extraction and supercritical carbon dioxide extraction each recover different groups of metabolites depending on solvent polarity, extraction temperature and extraction time [74,75].

Hydrodistillation remains the most widely used method for obtaining frankincense essential oils [76]. However, prolonged heating may result in hydrolysis or thermal degradation of oxygenated terpenoids. In contrast, supercritical CO_2_ extraction generally preserves thermolabile constituents and yields extracts enriched in diterpenes and triterpenoids [77,78]. For *B. carteri*, supercritical CO_2_ extraction was evaluated at 15, 20 and 25 MPa; temperatures of 40, 50 and 60 °C; and extraction times of 60, 90 and 120 min. The method showed greater selectivity than steam distillation for obtaining fractions enriched in octyl acetate [79]. In *B. serrata*, essential oils obtained by hydrodistillation, steam distillation and supercritical CO_2_ extraction showed different relative concentrations of α-pinene, α-thujene, camphene, β-pinene, myrcene, limonene, m-cymene and cis-verbenol. α-Pinene and some oxygenated constituents were particularly prominent in the hydrodistilled oil, whereas several monoterpene hydrocarbons were more abundant in the steam-distilled product [56]. Recent work on *B. papyrifera* used sequential subcritical and supercritical CO_2_ extraction at approximately 10, 20 and 25 MPa, with temperatures of 25 and 40 °C. The process produced chemically distinct oleoresin fractions, with reported overall yields of approximately 23.8% for subcritical extraction and 25.1% for supercritical extraction. Incensole was the dominant identified constituent in some fractions, reaching approximately 36% of the relative chromatographic peak area, while incensole acetate represented approximately 17–20%. Increasing pressure and extraction time increased recovery, and subcritical CO_2_ displayed faster extraction kinetics than the supercritical process [77].

Soxhlet and batch extraction have been used as reference procedures for extracting AKBA from *B. serrata*; in one comparison, optimized batch extraction required 120 min and produced 4.8 ± 0.04 mg/g AKBA, whereas ultrasound-assisted extraction (UAE) achieved 6.5 ± 0.05 mg/g in only 10 min [80]. In a separate optimization study targeting KBA, methanolic UAE performed better than microwave-assisted extraction; the reported optimum involved a solvent-to-drug ratio of 20.42 mL/g, temperature of 44.01 °C and a duration of 11.54 min, producing 8.44% KBA by weight [81]. Therefore, extraction parameters should be carefully optimized according to the intended application, whether for essential oil production, phytochemical characterization, or the recovery of bioactive compounds for pharmaceutical, cosmetic, or nutraceutical use [75].

## 5. Analytical Techniques for Chemical Profiling

Reliable authentication of *Boswellia* species and frankincense-derived products depends fundamentally on accurate chemical characterization. Analytical methodologies have evolved considerably during the last two decades. Early investigations relied primarily on gas chromatography coupled with flame ionization detection (GC-FID) or mass spectrometry (GC-MS) for volatile profiling and high-performance liquid chromatography (HPLC) for the quantification of boswellic acids [1,82]. More recently, advances in ultra-high-performance liquid chromatography (UHPLC), high-resolution mass spectrometry (HRMS), nuclear magnetic resonance (NMR) spectroscopy, comprehensive two-dimensional gas chromatography (GC × GC-MS), and ambient ionization mass spectrometry have substantially improved analytical sensitivity, metabolite coverage, and discriminatory power (Table 2) [36,46,63,83].

The selection of an analytical platform depends primarily on the analytical objective, including species discrimination, geographical origin verification, quality control, detection of adulteration, metabolite discovery, or standardization of pharmaceutical extracts.

GC-MS/SPME-GC-MS remained central for volatile chemotaxonomy, particularly because the relative abundance of monoterpenes, sesquiterpenes, diterpenes and unusual volatile markers can distinguish several *Boswellia* taxa. A comparative work by Niebler and Buettner [39] already demonstrated species separation from volatile profiles, while GC × GC-TOF-MS subsequently increased peak capacity and identification confidence for complex samples such as *B. papyrifera* [54]. The discovery and characterization of *B. occulta* is particularly instructive: GC-MS/GC-FID combined with NMR revealed a methoxyalkane-rich chemical phenotype that is strikingly different from the profiles of conventional traded frankincense species [16,34].

For essential oil authentication, conventional relative-abundance GC-MS profiles alone are not always sufficient because an adulterant can be selected to reproduce the apparent concentration of major terpenes. Recent Ojha et al.’s analysis of commercial *B. carteri* oils demonstrated by combined GC-MS/CGC-MS that enantiomeric distributions of α-pinene, limonene, sabinene, terpinen-4-ol and other monoterpenes represent highly reliable authentication markers capable of distinguishing authentic *B. carteri* essential oils from adulterated commercial products [13]. Chiral fingerprints have therefore emerged as a robust analytical tool for authenticity assessment of frankincense essential oils.

For the non-volatile resin fraction, HPLC remains the main quantitative reference platform because compounds, such as α-/β-boswellic acid, KBA, AKBA and related acetylated/tirucallic acids are much less suited to routine volatile analysis. HPLC-UV/DAD studies have therefore provided validated multi-analyte assays for resin extracts and later supported interspecies comparisons and chemometric classification [82]. Importantly, this also illustrates why AKBA or KBA alone should not be treated as universal species-authentication markers: their concentrations are strongly species-dependent, and broader chromatographic or spectroscopic fingerprints generally contain greater taxonomic information. The HPLC/NIR study directly demonstrated species-specific variation in these boswellic acids across seven species [63].

The most notable recent development is the shift toward rapid fingerprinting and chemometrics. MALDI-TOF-MS allowed rapid recognition of characteristic boswellic acid/triterpenoid ions in commercial products [84]. Ambient ionization mass spectrometry, including direct analysis in real time (DART-MS), enables rapid analysis of intact resin without chromatographic separation. A recent study successfully classified nine commercially important *Boswellia* species using DART-TOF-MS combined with chemometric analysis [36]. NIR spectroscopy represents an even more attractive routine-screening approach because intact solid resin can be analyzed non-destructively; when calibrated against HPLC and coupled with PCA/PLSR, it can provide both taxonomic information and estimates of important quality markers such as AKBA [63].

Untargeted metabolomics utilizing UHPLC-QTOF-MS, UHPLC-Orbitrap-MS and related platforms enables simultaneous detection of hundreds of metabolites without prior knowledge of sample composition. Li et al. [83] using UPLC-QTOF-MS/MS illustrated a further move from authentication of species toward process authentication and quality-state characterization. Untargeted/high-resolution MS combined with targeted quantification and chemometrics can determine whether traditional processing, extraction or manufacturing affects the metabolite profile, an issue that is increasingly important when authentic botanical starting material is converted into medicinal extracts or finished frankincense products.

NMR is especially important for the definitive structural elucidation of boswellic acids, diterpenoids and other non-volatile constituents isolated from *Boswellia* resins. Spectroscopic characterization of triterpenoids from *Boswellia* combined ^1^H/^13^C-NMR with IR and high-resolution MS to establish structures such as 3β,11β-dihydroxy-β-boswellic acid and boswellic aldehyde [46]. A recent comprehensive assessment compiled essentially complete ^13^C-NMR assignments for 119 of 127 reported *Boswellia* triterpenoids, encompassing ursane, oleanane, lupane, dammarane and tirucallane skeletons, and specifically highlighted these reference data as useful for the rapid identification and quality control of frankincense constituents [85].

The analytical techniques described above generate the chemical fingerprints upon which contemporary authentication strategies are based. Figure 3 summarizes analytical platforms for the chemical profiling of *Boswellia* species.

Overall, the analytical platforms are complementary rather than interchangeable. GC-MS and SPME-GC-MS are best suited to routine volatile profiling, whereas chiral GC-MS provides greater specificity for detecting essential oil adulteration through enantiomeric distributions. HPLC is comparatively accessible and robust for targeted quantification of boswellic acids, while high-resolution LC-MS provides broader metabolite coverage and higher identification confidence at greater cost and analytical complexity. MALDI-TOF-MS and DART-MS enable rapid fingerprinting with minimal sample preparation, although their quantitative performance and susceptibility to matrix effects can limit confirmatory applications. NIR is particularly suitable for rapid, non-destructive routine screening, but it depends on representative calibration models and generally provides less direct structural information. In contrast, NMR provides definitive structural elucidation and highly reproducible fingerprints, although its lower sensitivity, greater sample requirements and instrument cost restrict high-throughput use. Accordingly, rapid screening methods such as NIR or DART-MS are most effective when combined with confirmatory chromatographic, high-resolution MS or NMR analysis.

## 6. Authentication Strategies and Adulteration Detection

Authentication of *Boswellia* resins and derived frankincense products is analytically challenging because the term “frankincense” encompasses oleo-gum resins from several *Boswellia* species, while commercial materials may additionally occur as essential oils, powdered resins, standardized extracts, formulated dietary supplements, or mixtures. Authentication therefore requires more than the measurement of a single constituent. A robust strategy should establish: (i) botanical identity, i.e., whether the material originates from the claimed *Boswellia* species; (ii) chemical profile, including the presence and relative abundance of characteristic metabolites; (iii) product integrity, particularly the absence of undeclared *Boswellia* species, synthetic constituents, fillers, or compounds deliberately added to influence quality-control assays. No single analytical technique adequately addresses all these questions. Current evidence instead favors an orthogonal authentication strategy combining targeted chemical markers, chemical fingerprints, chemometric classification, genetic identification where feasible, and product-specific adulteration screening.

### 6.1. Marker-Based Chromatographic Authentication

Targeted chromatographic analysis remains one of the most established approaches for authenticating medicinal *Boswellia* products, particularly for crude resins and medicinal extracts. Pentacyclic triterpenoids, particularly α- and β-boswellic acids and their acetylated and 11-keto derivatives, are useful chemical indicators because their distributions vary among *Boswellia* resins and because several of these compounds are directly relevant to pharmacological standardization. Earlier comparative studies established that triterpenoid profiles obtained by TLC, HPLC, and LC–MS can distinguish certain *Boswellia* resins, although closely related or chemically similar materials cannot always be resolved using a small number of markers alone [26,86]. Chromatographic profiles can readily discriminate chemically distinctive species such as *B. frereana* from boswellic acid-rich resins, whereas differentiation of materials traded as *B. carteri* and *B. sacra* has historically been much more difficult because of their similar triterpenoid patterns.

Pharmacopeial specifications consequently provide an important, but relatively narrow, level of authentication. The USP monograph defines *B. serrata* as the oleo-gum resin of *B. serrata* and specifies not less than 1.0% keto derivatives of β-boswellic acid, calculated as the sum of KBA and AKBA on the dried basis [87]. Quantification of KBA, AKBA and related boswellic acids by validated HPLC or UHPLC methods is therefore valuable for testing the profile and potency of *B. serrata* extracts. However, meeting a numerical specification for one or two marker compounds does not by itself demonstrate botanical authenticity. Closely related species may share the same triterpenoid classes, whereas intentional enrichment, selective extraction, or the addition of non-*Boswellia* acidic compounds can affect apparent assay values.

The limitations of simple “total boswellic acid” assays were demonstrated particularly clearly by Püski et al. [88], who examined 14 commercial *B. serrata* extracts using HPLC, UHPLC-HRMS and ^1^H NMR. Only two samples fulfilled the expected KBA/AKBA criteria, while ten contained approximately 6–11% citric acid. Because titrimetric determination of “total boswellic acids” responds nonspecifically to acidic constituents, the added citric acid could artificially increase the apparent total-acid content without providing authentic boswellic acids. The study provides an important example of analytical-method-directed adulteration, in which an inexpensive adulterant can exploit the nonspecificity of a quality-control assay [88]. Consequently, standardization claims should preferably be verified by validated chromatographic quantification of defined boswellic acids rather than by nonspecific bulk-acidity measurements.

### 6.2. Metabolomic Fingerprints and Chemometric Classification

Untargeted or broadly targeted metabolomics provides a more powerful framework because authentication is based on a multivariate chemical profiling, rather than analyzing and comparing a single compound. LC–MS or LC–MS/MS profiling simultaneously captures boswellic acids, other triterpenoids, diterpenoids and numerous minor metabolites, after which principal component analysis (PCA), hierarchical clustering, partial least-squares methods or supervised classification algorithms can identify patterns associated with species, provenance or processing. Such approaches are particularly valuable when species share major marker compounds, but differ in combinations of less abundant metabolites.

A recent LC–MS/MS metabolomics investigation of *B. serrata*, *B. sacra*, *B. carteri* and *B. frereana* materials annotated 77 metabolites and demonstrated clear sample differentiation using chemometric analysis. Interestingly, the multivariate separation was strongly influenced by the geographical source of the oleo-gum resins, while extraction and fractionation also altered the recovered metabolite pattern [23]. These observations are highly relevant to authentication model development: a classifier constructed from a geographically restricted reference set may inadvertently learn provenance-related chemistry rather than species identity. Authentication databases should therefore contain authenticated specimens representing multiple populations, harvest seasons, resin grades and processing conditions. Biological and technological variation must be incorporated into model validation rather than treated solely as analytical noise.

Ambient ionization mass spectrometry is emerging as an attractive complement to conventional LC–MS because it can greatly reduce sample preparation and chromatographic analysis time. Price et al. [36] analyzed commercially relevant resins from nine *Boswellia* species by direct analysis in real time-of-flight mass spectrometry (DART-TOF-MS). Chemometric treatment of the positive-ion mass spectra produced effective taxonomic separation among the examined species, demonstrating that whole-fingerprint mass-spectral data can support rapid identification of frankincense resins in trade [36]. Although larger interlaboratory reference libraries and external validation are still needed before such classifiers can be considered universal, DART-MS illustrates the shift from “marker detection” toward pattern-recognition-based authentication.

A key advantage of metabolomic authentication is its capacity to detect unexpected components rather than only confirming predefined analytes. This is particularly relevant to complex dietary supplements, where adulterants may not be structurally related to boswellic acids. Kosolapov et al. [89] developed a CE-MS/MS method capable of simultaneously examining boswellic acids and a panel of non-steroidal anti-inflammatory drugs as potential adulterants of *B. serrata* preparations. Although NSAID adulteration was not detected in the tested supplements, CE-HRMS analysis revealed substantial variation in boswellic acid profiles and detected additional undeclared compounds, including citric and ascorbic acids [89]. This type of targeted-plus-untargeted workflow is particularly suitable for regulatory surveillance because it assesses both identity markers and chemically unrelated adulterants in the same analytical framework.

### 6.3. Authentication of Essential Oils by GC–MS and Chiral Analysis

Authentication strategies for frankincense essential oils must differ from those applied to resin extracts. Boswellic acids are high-molecular-weight, non-volatile triterpenoids and are therefore not appropriate primary markers for steam-distilled essential oils. Instead, essential oil authentication relies mainly on the qualitative and quantitative distribution of monoterpenes, sesquiterpenes and other volatile constituents using GC-FID and GC–MS. Species-specific or chemotype-associated volatile signatures can provide powerful discrimination; for example, detailed characterization of *B. occulta* revealed an unusual abundance of methoxyalkanes that clearly differentiated its essential oil from more conventional frankincense profiles [34].

The addition of chiral GC substantially strengthens authentication because enantiomeric ratios can distinguish naturally biosynthesized terpenes from inexpensive synthetic replacements whose stereochemical composition differs from that of genuine essential oil. Ojha et al. [13] applied conventional GC–MS together with chiral GC–MS to 21 commercial oils marketed as *B. carteri* and two laboratory-distilled reference oils. Their analysis identified extensive quality problems: commercial samples showed evidence consistent with the addition of or contamination by synthetic limonene and octyl acetate, castor oil, other *Boswellia* species, phthalates, and other low-cost materials. Six of the twenty-one commercial samples were classified as adulterated with synthetic limonene alone [13]. The study demonstrates that a conventional GC–MS profile is not always sufficient. An adulterant can reproduce the concentration of a major terpene, whereas enantiomeric composition provides an additional biosynthetic authenticity criterion.

### 6.4. Adulteration and Commercial Fraud

Commercial frankincense products are susceptible to several authenticity and quality problems, including interspecific admixture or incorrect botanical identity, the addition of non-*Boswellia* materials, synthetic compounds or cheaper oils, and inaccurate compositional or provenance claims (Table 3).

The findings summarized in Table 3 demonstrate that authenticity and quality issues in commercial frankincense vary according to product type and should not necessarily be interpreted as deliberate fraud. For essential oils, the detection of adulteration is particularly challenging when added materials reproduce or closely resemble the natural volatile profile. In such cases, conventional compositional analysis can be strengthened by chiral profiling to identify atypical enantiomeric patterns indicative of exogenous or synthetic inputs [13]. In *B. serrata* extracts and supplements, the principal concern is the discrepancy between declared standardization and analytically verified composition, highlighting the limitations of nonspecific “total boswellic acid” claims [88,89,90].

Geographical origin represents an additional authentication challenge because provenance can influence both chemical composition and commercial value. However, direct evidence of deliberate geographical mislabeling in commercial frankincense remains limited.

However, to translate the available analytical evidence into routine practice, a product-specific authentication workflow is proposed for raw resins, essential oils, extracts, and frankincense products (Figure 4). A practical authentication should begin by recording the claimed species, origin, product type, processing history and applicable quality specifications. The sample should then be assigned to a matrix-specific pathway because raw resin, essential oil, extract and products require different analytical approaches. Raw resins and extracts may first be assessed by targeted HPLC/UHPLC analysis of boswellic acids, followed by broader LC-MS, NIR or DART-MS fingerprinting and chemometric comparison with authenticated reference samples [26,33,63]. Because boswellic acid concentrations vary among species, compliance with a single-marker specification should not alone be interpreted as proof of botanical identity [63,87]. Essential oils should first be characterized by GC-MS and then examined by chiral GC-MS when adulteration is suspected. Enantiomeric distributions provide evidence complementary to relative-abundance profiles and can reveal the addition of synthetic terpenes or oils of another botanical origin [13]. Finished supplements should undergo both targeted measurement of declared markers and broader screening for unexpected constituents. Combined targeted and high-resolution MS analysis has detected substantial variation in boswellic acid profiles and undeclared compounds in commercial preparations [89].

The final decision should integrate the targeted assay, chemical fingerprint and reference comparison. A sample may be classified as authentic and compliant, authentic but outside specification, inconclusive, or misidentified/adulterated. Discordant results or samples outside the validated range of the reference model should undergo repeat or confirmatory analysis.

## 7. Emerging Technologies and Future Perspectives

Advances in metabolomics, high-resolution analytical platforms and computational analysis are expanding opportunities for the authentication of *Boswellia* species and frankincense products. While conventional chromatographic and spectroscopic methods remain important for routine quality control, future strategies are likely to combine comprehensive chemical fingerprints with chemometrics, molecular identification and quality markers.

### 7.1. Chemometrics and Machine Learning-Assisted Authentication

Metabolomic datasets contain large numbers of correlated variables and therefore require multivariate analysis to identify meaningful chemical patterns. Chromatographic fingerprinting combined with multivariate data analysis provides an effective strategy for authentication, as complex compositional information can be exploited for sample classification without relying exclusively on individual marker compounds [91]. Unsupervised approaches such as principal component analysis (PCA) and hierarchical cluster analysis (HCA) are useful for visualizing sample clustering, detecting outliers and identifying chemical differences associated with species or geographical origin. For example, LC–MS/MS metabolomic profiling combined with PCA, HCA and OPLS-DA has demonstrated substantial chemical differences between *Boswellia* species and enabled the differentiation of samples of various geographical origins [23].

Rapid mass-spectral fingerprinting also shows considerable promise. DART-TOF-MS combined with chemometric analysis successfully differentiated commercial resins representing nine *Boswellia* species, demonstrating its potential for taxonomic authentication of frankincense in trade [36].

Machine learning algorithms, such as Random Forest (RF), Support Vector Machines (SVMs), Artificial Neural Networks (ANNs), Extreme Gradient Boosting (XGBoost), and Convolutional Neural Networks (CNNs) could further improve classification and assist in identifying discriminatory metabolites. Machine learning is increasingly used for high-dimensional metabolomic data because it can identify complex relationships that may not be captured by conventional statistical approaches [92].

### 7.2. Integrated Molecular and Chemical Authentication

Chemical composition can vary with geographical origin, environmental conditions, harvesting and processing. Consequently, metabolomic fingerprints alone may not always provide unequivocal evidence of botanical identity. Combining DNA-based identification with metabolomics could provide a more robust authentication strategy: genetic methods establish species identity, whereas metabolomics assesses the expressed chemical phenotype and product quality.

A practical future workflow could therefore combine DNA-based identification, untargeted metabolomic fingerprinting and targeted determination of chemical markers. Transcriptomic approaches may additionally help clarify the biosynthetic basis of species-specific terpene and triterpenoid profiles, although such applications remain largely exploratory in *Boswellia*.

### 7.3. Reference Fingerprints and Marker Validation

A major limitation of current *Boswellia* authentication research is the lack of large, standardized reference datasets. Published studies vary in species coverage, geographical sampling, extraction procedures and analytical platforms, limiting direct comparison and external validation. The successful differentiation of nine *Boswellia* species by DART-TOF-MS illustrates the value of well-characterized reference collections [36].

Future reference libraries should include authenticated species, geographical origin, harvest information, sample-processing conditions, raw analytical data, annotated metabolites and quality markers. Such datasets should follow FAIR principles to facilitate data sharing, interlaboratory comparison and development of robust classification models.

### 7.4. Standardization and Regulatory Translation

Despite substantial analytical progress, internationally standardized authentication criteria remain better established for *B. serrata* than for other commercially important *Boswellia* species. The USP monograph for *B. serrata* oleo-gum resin specifies not less than 1.0% combined keto derivatives of β-boswellic acid, and a separate monograph addresses *B. serrata* extracts [87].

Although such targeted markers are valuable for quality control, they do not provide comprehensive authentication across the genus. Future standards should therefore incorporate authenticated botanical reference materials, validated species-specific chemical fingerprints, standardized analytical procedures and interlaboratory validation. Attention is required for commercially important species such as *B. sacra*, *B. papyrifera* and *B. frereana*, for which internationally standardized analytical specifications remain comparatively limited.

## 8. Conclusions

Frankincense represents one of the world’s oldest and most economically important natural products, yet its increasing commercial value has intensified challenges related to species authentication, adulteration, geographical origin verification, and quality assurance. Over the past two decades, advances in chromatographic, spectroscopic, and mass spectrometric techniques have substantially improved the chemical characterization of *Boswellia* species. More recently, metabolomics integrating multivariate data analysis has shifted authentication from single-marker analyses towards comprehensive chemical fingerprinting, enabling a more robust discrimination of species, detection of adulteration, and identification of high-quality commercial products. Despite these advances, significant challenges remain. Natural chemical variability, incomplete reference databases, taxonomic complexity, and the absence of standardized analytical standards continue to limit reproducibility and regulatory implementation. Future authentication strategies should therefore integrate untargeted metabolomics, multi-omics approaches, machine learning, digital traceability, and quality markers within internationally standardized analytical frameworks.

## Figures and Tables

**Figure 1:**
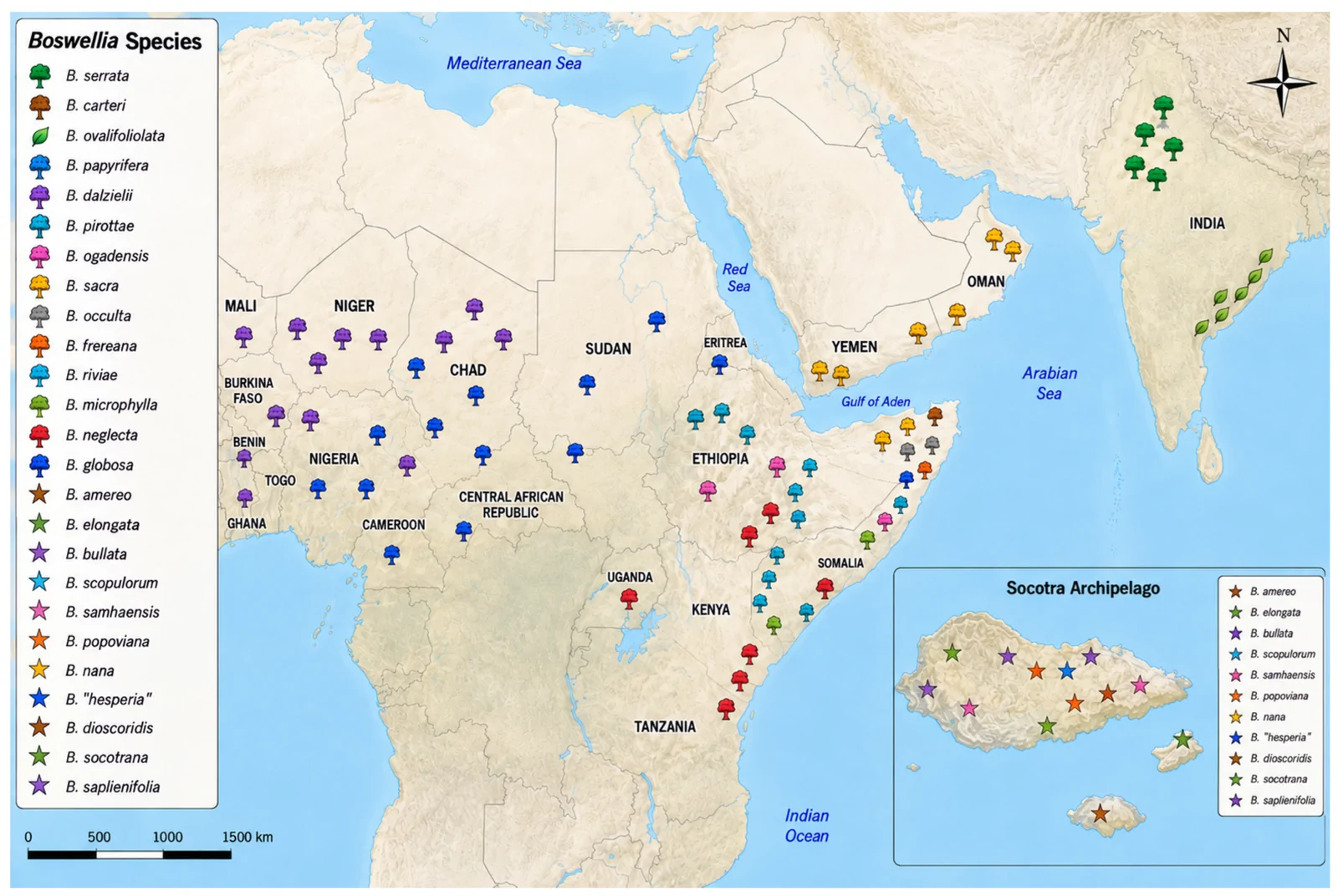
Geographical distribution of the *Boswellia* species. Presence by country/region [8,12].

**Figure 2:**
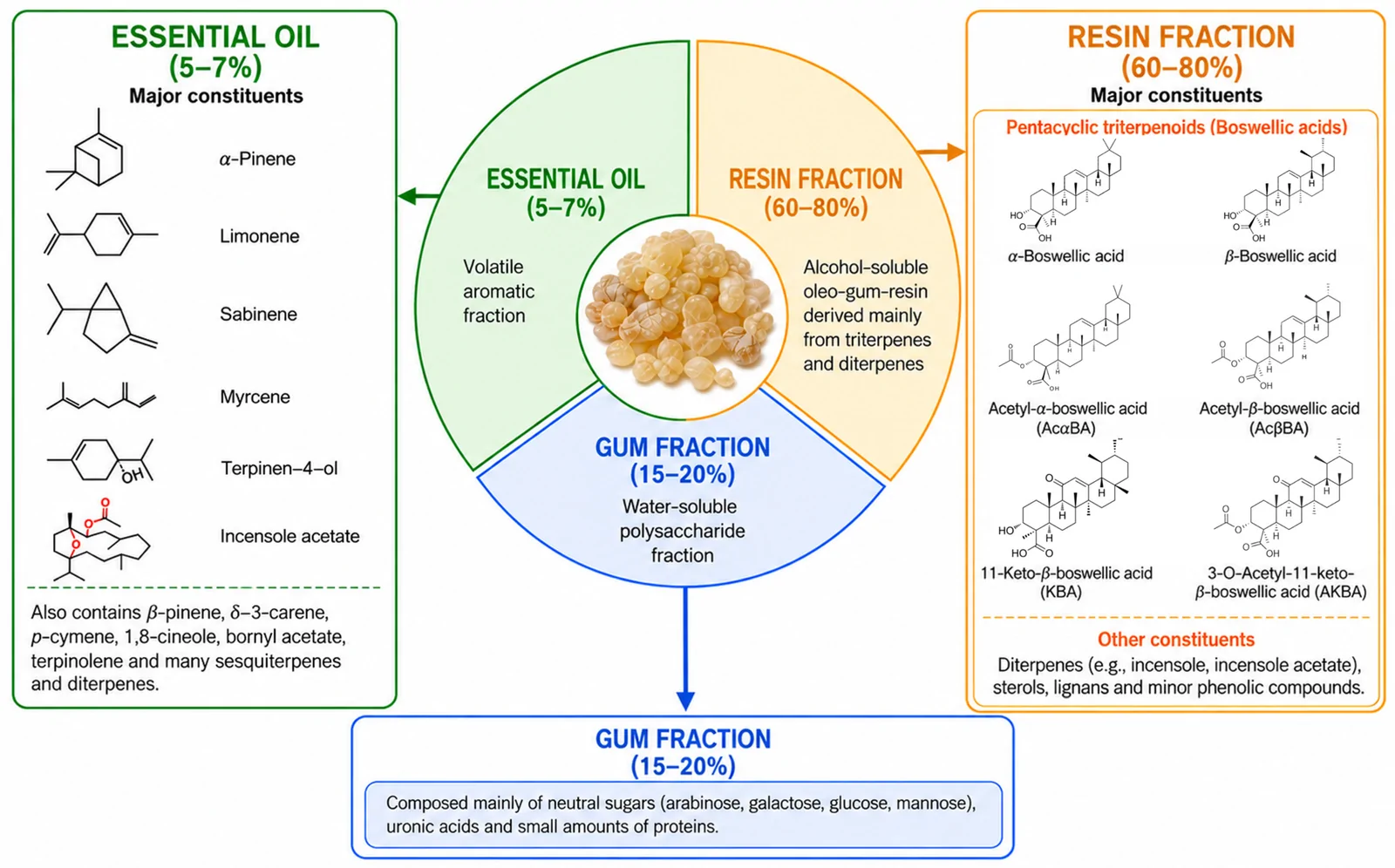
Chemical composition of frankincense resin.

**Figure 3:**
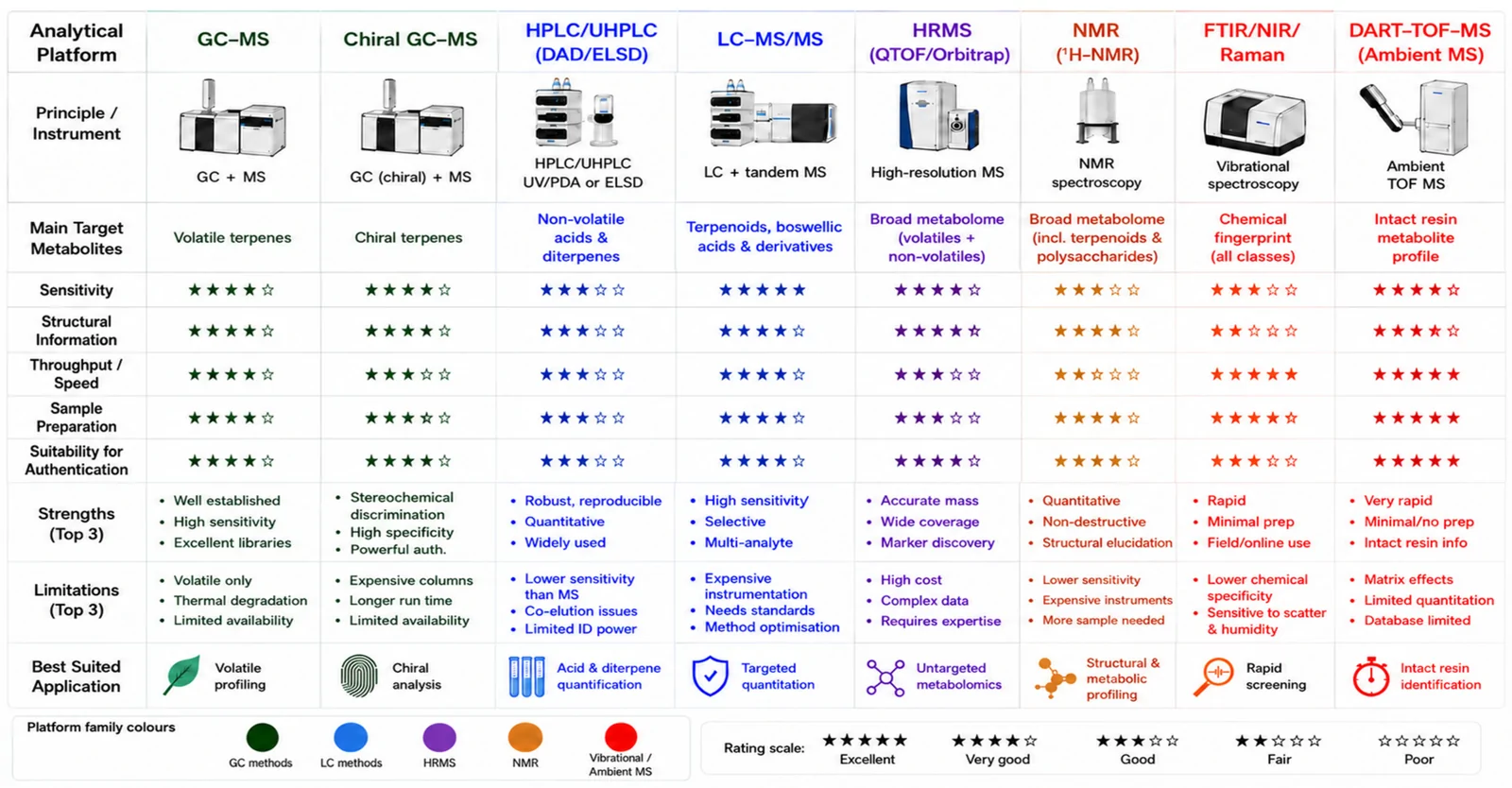
Comparison of analytical platforms for the chemical profiling of *Boswellia* species.

**Figure 4:**
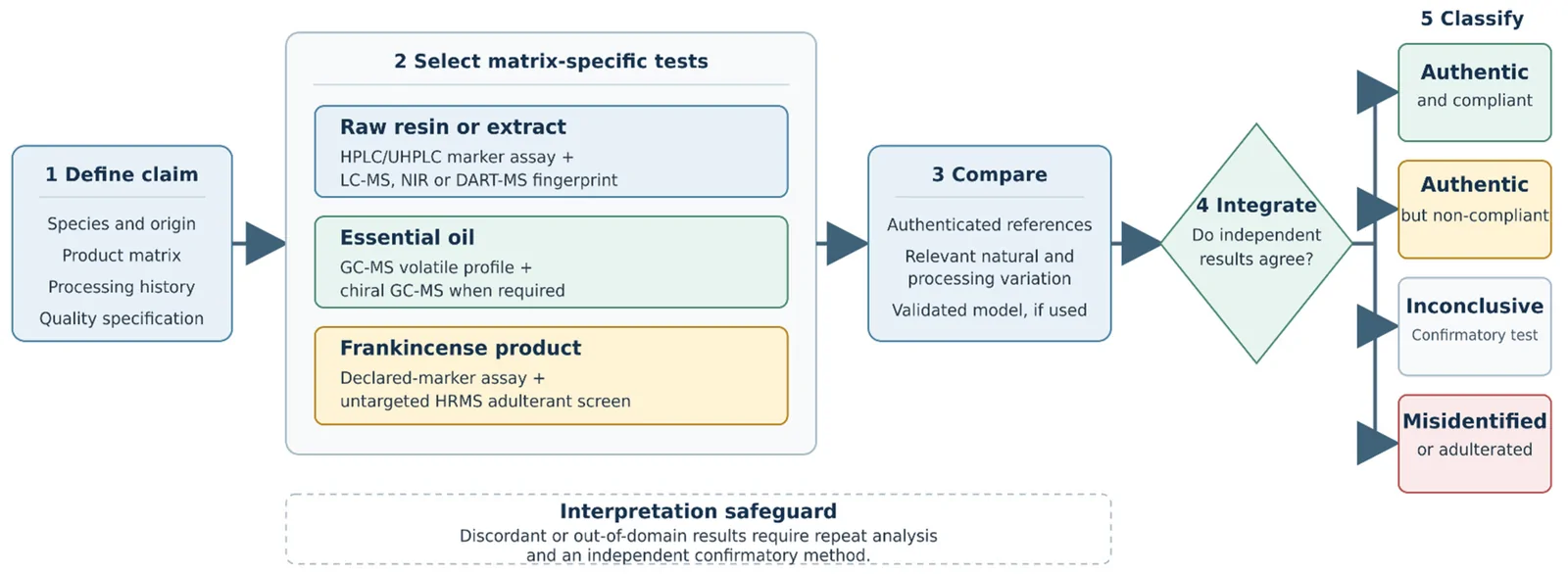
Proposed practical workflow for authentication of *Boswellia* resins and frankincense products. The workflow is a conceptual decision framework and requires matrix-specific and interlaboratory validation before regulatory implementation.

**Table 1:** Commercially important *Boswellia* species.

Taxon	Principal Distribution/ Commercial Origin	Commercial Product	Principal Commercial Uses	References
*B. sacra*	Oman, Yemen, northern and northeastern Somalia	Frankincense resin; essential oil	Incense, perfumery and cosmetics, essential oils, traditional medicine	[1,2]
*B. carteri*	Somalia, particularly Somaliland and Puntland	Frankincense resin; essential oil	Incense, aromatherapy, cosmetics, perfumery, medicinal/pharmacological products	[5,13]
*B. papyrifera*	Nigeria, Cameroun, Tchad, Central African Republic, Ethiopia, Eritrea, Sudan	Major internationally traded frankincense resin	Incense, essential oils, perfumery and industrial extracts	[6,25]
*B. serrata*	India	Indian frankincense resin; standardized *Boswellia* extracts	Herbal medicines, pharmaceutical preparations and dietary supplements	[6,26]
*B. frereana*	Northern Somalia	Maydi resin	Chewing resin, incense and perfumery	[1,6]
*B. neglecta* *	Ethiopia, Kenya, Somalia, northeastern Uganda, northeastern Tanzania	Resin (“black frankincense” in parts of the trade); essential oil	Incense, fragrance and essential oil products	[6,27]
*B. rivae* *	Ethiopia, Kenya and Somalia	Resin; essential oil	Incense, fragrance and emerging commercial products	[6,28]
*B. occulta* *	Somalia	Resin (historically traded as *B. carteri*)	Incense, essential oil production, authentication and traceability studies	[13,29]

* Moderate contributors to international frankincense production.

**Table 2:** Chemical constituents reported for commercially important *Boswellia* species.

*Boswellia* Species	Volatility Domain	Representative Identified Compounds	Chemical Class	Material or Fraction	Analytical Method	References
*B. sacra*	Volatile	α-Pinene; α-thujene; β-pinene; sabinene; camphene; myrcene; limonene; δ-3-carene; p-cymene; α-/β-phellandrene; γ-terpinene; terpinolene; (E)-β-ocimene	Monoterpene hydrocarbons	Hydrodistilled oleo-gum resin essential oil; headspace/resin oil	GC–FID; GC–MS; headspace/SPME–GC–MS	[30,39,40]
*B. sacra*	Volatile	Linalool; terpinen-4-ol; α-terpineol; borneol; trans-pinocarveol; cis-/trans-verbenol; verbenone; camphor; fenchone; pinocarvone	Oxygenated monoterpenes	Hydrodistilled/headspace volatile fraction of oleo-gum resin	GC–FID; GC–MS; headspace/SPME–GC–MS	[39,40]
*B. sacra*	Volatile/semi-volatile	(E)-β-Caryophyllene; α-copaene; β-elemene; germacrene D; α-humulene; cadinene derivatives; caryophyllene oxide; rotundone; mustakone	Sesquiterpene hydrocarbons and oxygenated sesquiterpenes	Resin essential oil/sensory-guided volatile fractions	GC–MS; preparative chromatography; MS; NMR for isolated trace odorants	[30,39,41]
*B. sacra*	Non-volatile	Boscartins L–O; incensole acetate; incensole oxide; incensole oxide acetate; isoincensole oxide; additional oxygenated cembranes	Cembrane-type diterpenoids	Organic-solvent resin fraction	Chromatographic isolation; HRMS/MS; 1D/2D NMR; X-ray	[42,43]
*B. sacra* (=*B. carteri* in Mannino et al.)	Non-volatile	α-Boswellic acid; β-boswellic acid; acetyl-α-boswellic acid; acetyl-β-boswellic acid; KBA; AKBA	Pentacyclic triterpenic acids	Lipophilic oleo-gum resin extract	HPLC–DAD; HPLC–DAD–ESI–MS/MS	[44]
*B. sacra*	Non-volatile	Lupeol; lupeolic acid; commic acid D; 9,11-dehydroboswellic acid; related ursane-, oleanane- and lupane-type compounds	Pentacyclic triterpene alcohols/acids	Organic-solvent resin extract	HPLC; MS/HREIMS/HRMS; ^1^H and ^13^C NMR; 2D-NMR; HPLC-DAD-MS/MS	[2,45,46]
*B. carteri*	Volatile	α-Pinene; α-thujene; β-pinene; sabinene; limonene; myrcene; p-cymene; camphene; (E)-β-ocimene	Monoterpene hydrocarbons	Commercial resin essential oil	GC–FID; GC–MS	[47]
*B. carteri*	Semi-volatile/non-volatile	Cembrene A; (3S,4S,7R)-4-hydroxycembrane; pavidolide D; 4-methoxy-5-hydroxycembrane; 4-hydroxycembra-5,8,12,15-tetraene	Cembranoid diterpenes	Organic-solvent gum-resin extract	Silica-gel/preparative chromatography; HRMS; 1D/2D NMR	[48]
*B. carteri*	Non-volatile	Boswellianols A–I; boscartin D; boscartol O; cneorubin A	Prenylmaaliane-, prenylaromadendrane-, and highly oxidized cembrane-type diterpenoids	Polar methanolic fraction after sequential petroleum-ether/methanol extraction	Silica gel; MPLC; semi-preparative HPLC; HR-ESI-MS; 1D/2D NMR; ECD	[49]
*B. carteri*	Non-volatile	α-/β-boswellic acids and acetylated/keto boswellic acids	Pentacyclic triterpenic acids	Lipophilic oleo-gum resin extract	HPLC–DAD; LC–MS(/MS)	[50]
*B. frereana*	Volatile	α-Thujene; α-pinene; sabinene; p-cymene; β-pinene; α-phellandrene; limonene; myrcene	Monoterpene hydrocarbons	Hydrodistilled oils from authenticated single-tree resin samples	GC–FID; GC–MS	[39,51]
*B. frereana*	Non-volatile	Lupeol; α-amyrin; 3-epi-lupeol; β-amyrin	Pentacyclic triterpene alcohols	Dichloromethane extracts of authenticated single-tree oleo-gum resins	GC–MS	[51]
*B. frereana*	Non-volatile	α-Amyrin acetate/related acetylated triterpenes	Acetylated pentacyclic triterpenes	Dichloromethane resin extract	GC–MS	[51]
*B. papyrifera*	Volatile	Octyl acetate; octanol; limonene; (E)-β-ocimene; smaller monoterpenes	Aliphatic ester/alcohol, monoterpenes	Hydrodistilled oils from authenticated resin samples	GC–MS; GC–FID; hierarchical clustering	[52,53]
*B. papyrifera*	Volatile/semi-volatile	Numerous mono-, sesqui- and diterpenoid volatiles, including cembrane/verticillane constituents	Mixed volatile terpenoids	Volatile/semi-volatile oleo-gum resin fraction	GC × GC–TOF-MS with 2D retention indices	[54]
*B. papyrifera*	Semi-volatile	Cembrene A; verticilla-4(20),7,11-triene; incensole; incensyl/incensole acetate	Cembrane-/verticillane-type diterpenoids	Essential oil semi-volatile fraction	GC–MS; GC–FID; GC × GC–accurate-mass TOF–MS	[52,53,54]
*B. papyrifera*	Non-volatile	α-Amyrin; β-amyrin; α-amyrenone; β-amyrenone; α-boswellic acid; nortriterpenes including 24-noroleana-/24-norursa-3,12-diene	Pentacyclic triterpenes, ketones, acids and nortriterpenes	Methanol resin extract	GC; GC–MS	[52]
*B. serrata*	Volatile	α-thujene; α-pinene; β-pinene; sabinene; limonene; myrcene; p-cymene; oxygenated monoterpenes including terpinen-4-ol, linalool, α-terpineol, borneol, verbenol	Monoterpenes	Hydrodistilled oleo-gum resin essential oil	GC–MS	[55,56]
*B. serrata*	Volatile/semi-volatile	β-Caryophyllene; α-copaene; germacrene D; β-elemene; nerolidol; caryophyllene oxide; spathulenol	Sesquiterpenes and oxygenated sesquiterpenes	Hydrodistilled oleo-gum resin essential oil	GC–MS	[55]
*B. serrata*	Semi-volatile/non-volatile	Cembrene A; thunbergene; verticilla-4(20),7,11-triene; incensole; cembra-2,7,11-trien-4,5-diol; cembrenol and related oxygenated cembranoids	Diterpene hydrocarbons and oxygenated cembranoids	n-Hexane gum extract and/or oil semivolatile fraction	GC–MS	[55]
*B. serrata*	Non-volatile	α-Boswellic acid; β-boswellic acid; acetyl-α-boswellic acid; acetyl-β-boswellic acid; KBA; AKBA; keto-/acetyl-tirucallic acids	Pentacyclic boswellic acids and tirucallane-type triterpenoids	Ethanolic/petroleum ether/lipophilic gum-resin extracts	HPLC–UV/DAD; HPLC–DAD–ESI–MS/MS; LC–MS(/MS)	[57]
*B. serrata*	Non-volatile	Lupeol; α-amyrin; β-amyrin and related neutral triterpenes	Pentacyclic triterpene alcohols	Nonpolar/methanolic gum-resin extract	GC–MS; MS/NMR	[55,58]
*B. serrata*	Non-volatile	24-norursa-3,12-diene; 24-norursa-3,12-dien-11-one and related nonpolar constituents	Nortriterpenes	n-Hexane gum extract	GC–MS	[55]
*B. neglecta*	Volatile	α-Thujene; α-pinene; β-pinene; sabinene; limonene; p-cymene; γ-terpinene; terpinen-4-ol; linalool; α-terpineol; trans-pinocarveol; verbenol	Mono-/oxygenated monoterpenes	Hydrodistilled oil or headspace volatile fraction of authenticated resin	GC–FID; GC–MS	[52]
*B. neglecta*	Non-volatile	α-Amyrin; β-amyrin; α-amyrenone; β-amyrenone; related triterpenes	Pentacyclic triterpene alcohols and ketones	Methanol resin extract	GC; GC–MS	[52]
*B. rivae*	Volatile	α-Thujene; α-pinene; β-pinene; δ-3-carene; p-cymene; limonene; β-phellandrene	Monoterpene hydrocarbons	Hydrodistilled resin oils	GC–FID; GC–MS	[28,53]
*B. rivae*	Volatile/semi-volatile	trans-Pinocarveol; trans-verbenol; terpinen-4-ol; α-terpineol; trans-β-elemene; β-caryophyllene; α-copaene	Oxygenated monoterpenes and sesquiterpenes	Hydrodistilled oleo-gum resin essential oil	GC–FID; GC–MS	[28]
*B. rivae*	Non-volatile	α-Amyrin; β-amyrin; α-amyrenone; β-amyrenone; related triterpenes	Pentacyclic triterpene alcohols/ketones and nortriterpenes	Methanol resin extract	GC; GC–MS	[52]
*B. occulta*	Volatile	1-Methoxyhexane; 1-methoxyheptane; 1-methoxyoctane; 1-methoxynonane; 1-methoxydecane; 1-methoxyundecane	Long-chain methyl ethers/methoxyalkanes	Hydrodistilled essential oils from 12 authenticated single-tree resin samples	GC–FID; GC–MS	[16]
*B. occulta*	Volatile	α-Thujene; α-pinene; camphene; sabinene and minor monoterpenes	Monoterpene hydrocarbons	Hydrodistilled essential oils from 12 authenticated single-tree resin samples	GC–FID; GC–MS	[16]
*B. occulta*	Volatile/semi-volatile	α-Copaene; β-caryophyllene; β-elemene; 4,10-di-epi-guaiol; guaiol; serratol	Sesquiterpene hydrocarbons, oxygenated sesquiterpenes and diterpene alcohols	Hydrodistilled essential oils from 12 authenticated single-tree resin samples	GC–FID; GC–MS	[16]

**Table 3:** Adulterants and authenticity/quality issues in commercial frankincense products.

Reported Adulterant/ Authenticity Issue	Commercial Product Examined	Analytical Approach	Diagnostic Basis/Finding	Reference
Species mislabeling/incorrect botanical identity	Commercial gum resins and essential oils labeled as *B. sacra*, *B. serrata*, *B. papyrifera* or *B. frereana*	SPME–GC–MS/GC–MS	216 compounds were identified or tentatively identified. Few exceptions were detected within declared identities.	[39]
*B. occulta* oil/material	Commercial oils labeled *B. carteri*	GC–MS; CGC–MS	Two samples were contaminated with *B. occulta*; octyl methyl ether and/or decyl methyl ether were discussed as indicators associated with *B. occulta*.	[13]
*B. serrata* oil/material	Commercial oils labeled *B. carteri*	GC–MS; CGC–MS	Comparison with authentic *B. carteri* profiles identified one sample contaminated/adulterated with *B. serrata*.	[13]
Synthetic limonene	*B. carteri* essential oils	GC–MS; CGC–MS	Comparison of 21 commercial oils with two laboratory-distilled oils identified six commercial samples as adulterated with synthetic limonene; chiral-terpenoid distributions provided additional authentication information.	[13]
Synthetic octyl acetate	*B. carteri* essential oils	GC–MS; CGC–MS	Three commercial samples were reported contaminated with synthetic octyl acetate.	[13]
Castor oil	*B. carteri* essential oils	GC–MS; CGC–MS	Chemical profiling of commercial oils identified three samples adulterated with castor oil.	[13]
Frankincense resin	*B. carteri* essential oils	GC–MS; CGC–MS	Two commercial samples were reported contaminated with frankincense resin; methyl commate-related compounds were used as evidence of resin addition.	[13]
Copaiba resin	*B. carteri* essential oils	GC–MS; CGC–MS	Trace copalic acid in sample indicated addition of copaiba resin; this sample also showed other adulteration/contamination.	[13]
Phthalates	*B. carteri* essential oils	GC–MS; CGC–MS	One commercial sample was reported contaminated with phthalates.	[13]
Cheap essential oil with similar chemical composition	*B. carteri* essential oils	GC–MS; CGC–MS	One sample was reported contaminated with another inexpensive essential oil of similar composition; enantiomeric profiling provides authentication information beyond conventional compositional analysis.	[13]
Citric acid	*B. serrata* extracts used in food supplements	HPLC; targeted UHPLC–HRMS; ^1^H NMR	Ten of fourteen commercial extracts contained approximately 6–11% citric acid, which is not characteristic of *B. serrata* extract. The addition could artificially increase apparent “total boswellic acid” values determined by acid–base titration.	[88]
Non-compliant KBA/AKBA content	*B. serrata* extracts used in food supplements	HPLC; UHPLC-HRMS	Only 2 of 14 investigated extracts fulfilled the expected KBA/AKBA specifications, demonstrating that nominal “total boswellic acid” values may not accurately represent concentrations of defined boswellic acids.	[88]
Undeclared citric and ascorbic acids/unexpected constituents	*B. serrata* preparations	CE-HRMS; CE-MS/MS	HRMS screening revealed substantial compositional variability and undeclared constituents	[89]
Potential NSAID adulteration investigated; non detected	*B. serrata* dietary supplements	Validated CE-MS/MS	The method targeted potential NSAID adulterants; application to commercial dietary supplements found no evidence of NSAID adulteration. This represents targeted surveillance rather than confirmed adulteration.	[89]
Low/inconsistent boswellic acid content	Commercial capsules, tablets, powders, gels and tea formulations containing *B. serrata*	UPLC-PDA; UPLC-Q-TOF-MS/MS	Wide variation in six boswellic acid markers was observed. Only four commercial formulations met the cited pharmacopeial limits for KBA and AKBA.	[90]

## Data Availability

No new data were created or analyzed in this study. Data sharing is not applicable to this article.
